# Supplementary material for: The Systems Biology Research Tool: evolvable open-source software
Source: BMC Syst Biol. 2008 Jun 29;2:55. doi: 10.1186/1752-0509-2-55 (PMC2446383; doi:10.1186/1752-0509-2-55)
Supplement: Additional file 1 — SBRT Archive. An archive of the current version of the Systems Biology Research Tool. [file 1752-0509-2-55-S1.zip › sbrt-1.4.0/doc/developers_guide/api/sbrt/ext/R/JriException.html]

JriException


|  |  |  |
| --- | --- | --- |
| **Overview** \| **Package** \| **Class** \| **Use** \| **Tree** \| **Deprecated** \| **Index** \| **Help** | |  |
| **PREV CLASS**   **NEXT CLASS** | **FRAMES**    **NO FRAMES**     **All Classes** |  |
| SUMMARY: NESTED \| FIELD \| CONSTR \| METHOD | DETAIL: FIELD \| CONSTR \| METHOD |  |


---


## sbrt.ext.R Class JriException

```
java.lang.Object
  java.lang.Throwable
      java.lang.Exception
          java.lang.RuntimeException
              sbrt.ext.R.JriException
```

**All Implemented Interfaces:**: java.io.Serializable

---

``` public class JriException extends java.lang.RuntimeException ```

This class is used to wrap exceptions thrown by the JRI.

**Author:**
:   This class was written and documented by
    Jeremiah Wright while in the Wagner lab.

**See Also:**: Serialized Form

---

**Constructor Summary**

`JriException(java.lang.String message,
java.lang.Throwable cause)`
  
          Constructs a JRI exception.

`JriException(java.lang.Throwable cause)`
  
          Constructs a JRI exception.


**Method Summary**

**Methods inherited from class java.lang.Throwable**

`fillInStackTrace, getCause, getLocalizedMessage, getMessage, getStackTrace, initCause, printStackTrace, printStackTrace, printStackTrace, setStackTrace, toString`

**Methods inherited from class java.lang.Object**

`clone, equals, finalize, getClass, hashCode, notify, notifyAll, wait, wait, wait`

**Constructor Detail**

### JriException

```
public JriException(java.lang.Throwable cause)
```

:   Constructs a JRI exception.

    **Parameters:**: `cause` - the original exception throw by the JRI.

---


### JriException

```
public JriException(java.lang.String message,
                    java.lang.Throwable cause)
```

:   Constructs a JRI exception.

    **Parameters:**: `message` - a descriptive message useful for debugging.: `cause` - the original exception throw by the JRI.


---


|  |  |  |
| --- | --- | --- |
| **Overview** \| **Package** \| **Class** \| **Use** \| **Tree** \| **Deprecated** \| **Index** \| **Help** | |  |
| **PREV CLASS**   **NEXT CLASS** | **FRAMES**    **NO FRAMES**     **All Classes** |  |
| SUMMARY: NESTED \| FIELD \| CONSTR \| METHOD | DETAIL: FIELD \| CONSTR \| METHOD |  |


---
